# Exploring causal correlations between inflammatory cytokines and coronary heart disease: A Mendelian randomization study

**DOI:** 10.1097/MD.0000000000039789

**Published:** 2024-10-11

**Authors:** Luo Lv, Yuli Guo, Zhongyi Zheng, Bao Li

**Affiliations:** a Department of Cardiology, The Second Hospital of Shanxi Medical University, School of Medicine, Shanxi Medical University, Taiyuan, China; b Department of Cardiology, The Frist Hospital of Shanxi Medical University, School of Medicine, Shanxi Medical University, Taiyuan, China; c Department of Urology, The First Hospital of Shanxi Medical University, School of Medicine, Shanxi Medical University, Taiyuan, China.

**Keywords:** coronary heart disease, GWAS, inflammatory cytokines, Mendelian randomization, single nucleotide polymorphisms

## Abstract

Coronary heart disease (CHD) is a global health concern, with inflammation significantly contributing to its pathogenesis. It is crucial to understand the relationship between inflammatory cytokines and CHD. This study investigates the causal correlations between circulating inflammatory cytokines and CHD using Mendelian randomization (MR), assessing both causative and resultant roles of these cytokines in CHD. In this bidirectional MR analysis, we used genetic data from a genome-wide association study (GWAS) of 60,801 CHD cases and 123,504 controls of European ancestry. We derived inflammatory cytokine data from a GWAS summary of 14,824 participants. The primary analytical approach was the inverse variance-weighted (IVW) method, supported by MR-Egger, weighted median, and weighted mode analyses. Heterogeneity was assessed using the Cochrane *Q* test, and horizontal pleiotropy was evaluated through the MR-Egger intercept and the MR-PRESSO global test, ensuring robustness against potential pleiotropic bias. This study pinpointed several cytokines as key upstream influencers on the risk of CHD, including eotaxin (CCL11) (odds ratio [OR]: 1.10, 95% confidence interval [CI]: 1.03–1.18, *P* = .003), C–C motif chemokine ligand 20 (CCL20) (OR: 1.15, 95% CI: 1.05–1.25, *P* = .002), macrophage colony-stimulating factor 1 (CSF1) (OR: 1.09, 95% CI: 1.01–1.17, *P* = .020), Fibroblast growth factor 21 (FGF21) (OR: 1.14, 95% CI: 1.01–1.29, *P* = .038), Fms-related tyrosine kinase 3 ligand (FLT3LG) (OR: 1.26, 95% CI: 1.09–1.44, *P* = .001), neurotrophin-3 (NT-3) (OR: 1.12, 95% CI: 1.01–1.24, *P* = .026), and leukemia inhibitory factor (LIF) (OR: 0.89, 95% CI: 0.80–0.99, *P* = .029). Conversely, T-cell surface glycoprotein CD5 (CD5) (beta: −0.15, 95% CI: −0.29 to −0.01, *P* = .042) were identified as downstream factors impacted by CHD. No evidence of heterogeneity or horizontal pleiotropy was detected across all results, and a leave-one-out analysis substantiated the robustness of these findings. These findings suggest that CCL11, CCL20, CSF1, FGF21, FLT3LG, NT-3, and LIF may play a crucial role in the pathogenesis of CHD. Additionally, CHD may impact the expression of CD5. Additional research is needed to explore the potential of these biomarkers in the prevention and treatment of CHD.

## 1. Introduction

Coronary heart disease (CHD) denotes the cardiac condition resulting from myocardial ischemia and hypoxia, attributable to coronary atherosclerosis.^[1]^ Despite substantial progress in its treatment, CHD remains a leading cause of global mortality and morbidity, posing significant challenges to public health systems.^[2,3]^ This scenario accentuates the imperative for continuous and multifaceted research into its etiology. Inflammation is a key component in the development of CHD,^[4]^ persistently present throughout the entire course of atherosclerosis.^[5,6]^ Observational studies have demonstrated an association between elevated levels of inflammatory markers such as interleukin-6 (IL-6), interleukin-18 (IL-18), matrix metalloproteinase-9 (MMP-9), TNF-α, and soluble CD40 ligand (sCD40L) with CHD.^[7–10]^ But the interpretation of their findings is often muddled by confounding factors, such as reverse causality and the influence of variables such as lifestyle choices, genetic predispositions, and concurrent medical conditions.

Mendelian randomization (MR) is a method in epidemiology that uses genetic variants as instruments to infer causal relationships between potentially modifiable exposures and health outcomes.^[11]^ The method leverages the fact that genetic variants are fixed at conception and are not affected by environmental or lifestyle factors that often confound observational studies.^[12]^ This method can provide more reliable evidence about whether an exposure is causally related to an outcome. We conducted a 2-sample bidirectional MR analysis to investigate the potential relationship between circulating inflammatory cytokines and CHD, along with determining the direction of causality in this association. Initially, we obtained validated genetic instrumental variables for 91 inflammatory cytokines from genome-wide association study (GWAS) data. Subsequently, we assessed their connections with CHD and delved into causality by reversing exposures and outcomes. Our study findings lend support to the idea of targeting specific inflammatory cytokines as a preventive strategy against CHD.

## 2. Materials and methods

This MR analysis utilized publicly available GWAS, and comprehensive details regarding the studies used can be found in Table S1, Supplemental Digital Content, http://links.lww.com/MD/N690. All the included studies underwent approval processes by their respective institutional review boards and ethical committees, and participants in these studies had provided signed consent forms.

### 2.1. Study design

**Figure 1.**
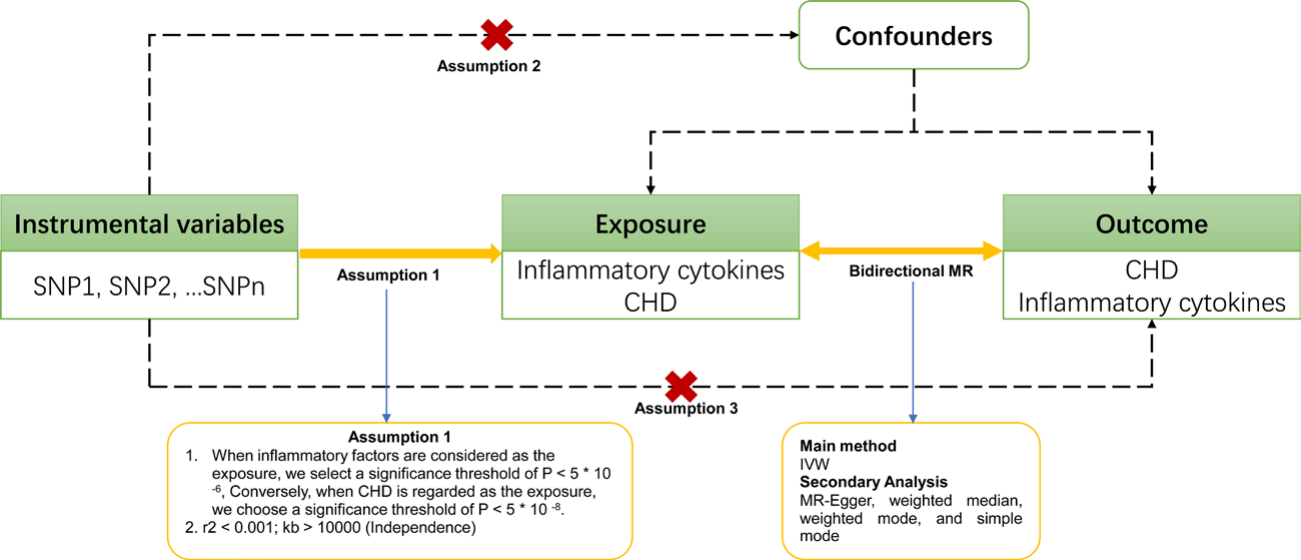
Flowchart illustrating the Mendelian randomization (MR) study on the causal link between 91 inflammatory cytokines and CHD. CHD = coronary heart disease; IVW = inverse variance weighted; SNP = single Nucleotide Polymorphism.

The study design overview is depicted in Figure 1. The MR analysis needs to satisfy 3 assumptions.^[13]^ Firstly, the relevance assumption stipulates that genetic variants serving as instrumental variables must have a robust association with the exposure. Secondly, the independence assumption ensures that these genetic variants are not linked to confounders that could influence both exposure and outcome, thereby avoiding confounding bias prevalent in observational studies. Lastly, the exclusion restriction mandates that genetic variants influence the outcome solely via their impact on the exposure, crucial for a causal interpretation of this relationship. In this bidirectional study, 2 separate GWAS datasets were employed to identify significant SNPs associated with 91 inflammatory cytokines and CHD. We initially designated each cytokine as a genetic instrumental variable to examine its potential causal relationship with CHD. Subsequently, genetic instrumental variables related to CHD were used to investigate causality with each inflammatory cytokine.

### 2.2. Data sources for inflammatory factors and coronary heart disease

The MR analysis utilizes publicly available GWAS summary data. For inflammatory cytokines, we selected the most comprehensive GWAS, which includes genome-wide associations of genetic variations for 91 inflammatory cytokines in 14,824 individuals of European ancestry.^[14]^ The CHD dataset utilized in this study was sourced from the CARDIoGRAMplusC4D consortium, encompassing 60,801 case subjects and 123,504 control subjects.^[15]^ The diagnostic criteria for CHD encompass myocardial infarction, acute coronary syndrome, chronic stable angina, or a coronary artery narrowing of more than 50%. It is essential to emphasize that the participant cohorts in the 2 GWAS datasets utilized in our investigation were entirely separate, ensuring no shared individuals across these datasets.

### 2.3. Instrumental variable selection

In our study, we initially established a genome-wide significance threshold at *P* < 5 × 10^−8^ to identify SNPs that are significantly associated with CHD and inflammatory cytokines. However, due to the scarce number of SNPs associated with inflammatory cytokines when considered as exposures, we revised this threshold to a more relaxed level of *P* < 5 × 10^−6^.^[16,17]^ To address potential issues of linkage disequilibrium, we employed a strategy of SNP clumping with parameters set to a 10,000 kb window and a *r*^2^ threshold of 0.001. In addition, all palindromic SNPs were systematically excluded from our analysis due to the ambiguity in their alignment direction in GWAS pertaining to systemic inflammatory regulators. We further evaluated the association strength between these genetic markers and inflammatory cytokines using their respective R^2^ values. Finally, the robustness of the instrumental variables was assessed using the *F*-statistic, with our analysis revealing strong instrumental variables as evidenced by an *F*-statistic exceeding 10, thereby confirming the minimal presence of weak instrument bias in our study.^[18]^

### 2.4. Statistical analysis

In our MR study, designed to uncover the genetic associations between 91 inflammatory cytokines and CHD outcomes, we utilized a variety of statistical methods to ensure a thorough and comprehensive analysis. The cornerstone of our approach was the random-effects inverse variance weighted (IVW) method, with each SNP being considered a reliable instrument.^[19]^ Mindful of the inherent limitations of this assumption, we incorporated additional methodologies as complementary tools to bolster analytical accuracy. These included MR-Egger, weighted median, weighted mode, and simple mode.^[20,21]^ Notably, the weighted median method proved invaluable in cases where a majority of the data stemmed from valid instruments.^[21]^ To rigorously assess heterogeneity across SNPs, we utilized Cochran *Q* test.^[22]^ Subsequently, we refined our findings by accounting for horizontal pleiotropy using the MR-Egger intercept and MR-PRESSO methods, thereby enhancing the robustness of our results.^[23]^ MR-Egger was particularly crucial for detecting unobserved pleiotropy and providing adjusted estimates, albeit with some loss in precision.^[20]^ The MR-PRESSO method was instrumental in identifying and correcting outliers among SNPs, thereby aligning the results with those obtained via the IVW approach.^[24]^ Additionally, we performed a “leave-one-out” analysis to investigate if the inferred causal link between exposure and outcome was disproportionately driven by any individual SNP. In this context, a *P*-value exceeding 0.05 suggested an absence of horizontal pleiotropy in our findings.^[25]^ To address the challenges of multiple testing, we applied the Bonferroni method. This strategy helped control the false discovery rate, distinguishing associations as either suggestive or significant based on an adjusted *P*-value of 0.00054 (0.05/91). All these analyses were performed using the two sample MR,^[26]^ Mendelian randomization,^[27]^ and MR-PRESSO R packages^[24]^ in R software versions 4.3.1, adhering to the rigorous standards of MR methodology.

## 3. Results

### 3.1. Effect of 91 inflammatory cytokines on CHD

**Figure 2.**
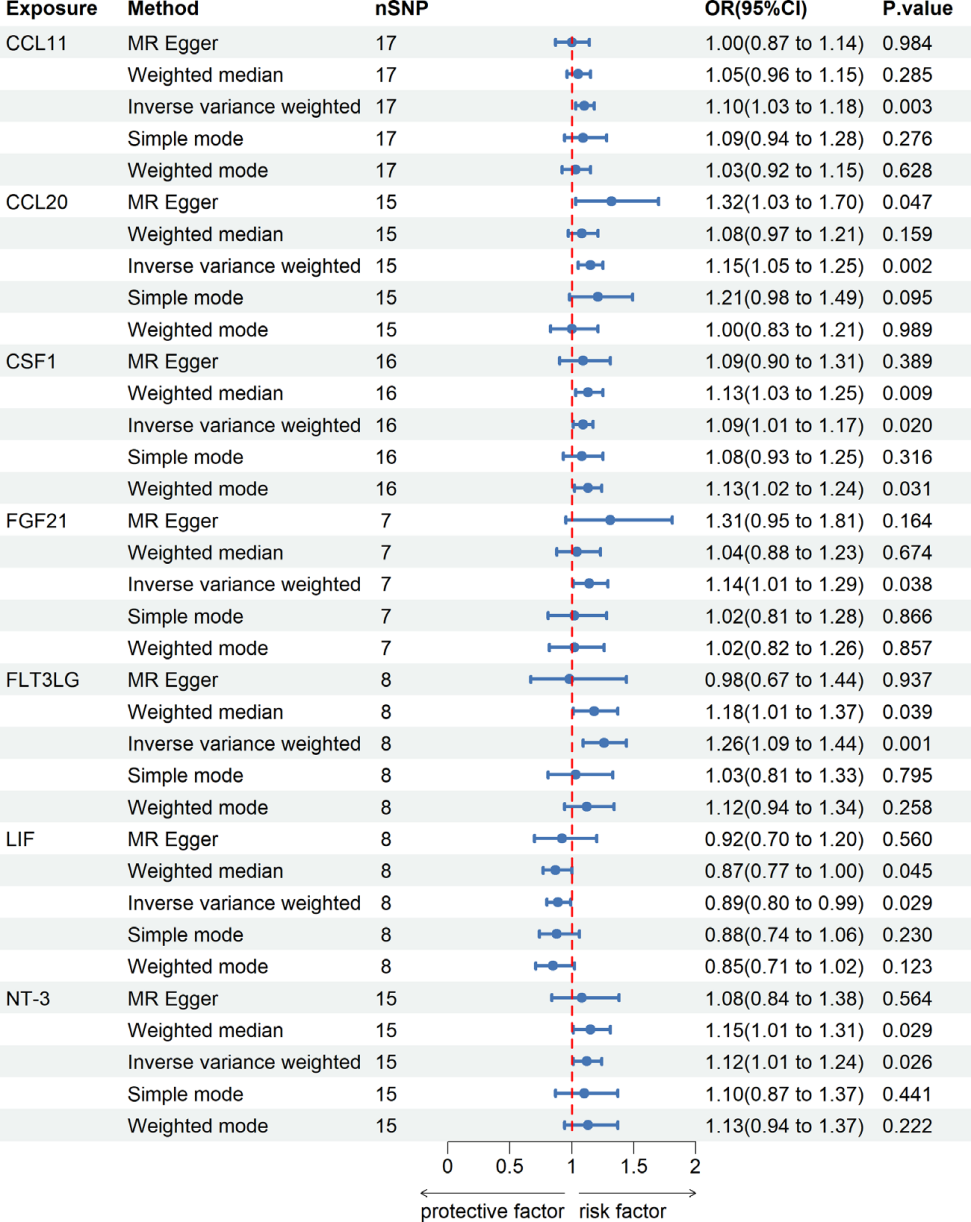
The causal effects of positive inflammatory cytokines on CHD using 5 MR methods. CCL11 = eotaxin levels; CCL20 = C–C motif chemokine 20 levels; CSF1 = macrophage colony-stimulating factor 1 levels; FGF21 = fibroblast growth factor 21 levels; FLT3LG = Fms-related tyrosine kinase 3 ligand levels; LIF = leukemia inhibitory factor levels; NT-3 = neurotrophin-3 levels.

In our examination of inflammatory factors for MR analysis, we faced a limitation in the number of valid SNPs due to the stringent genome-wide significance threshold set at 5 × 10^−8^. To ensure an adequate pool of SNPs for our MR study, we adopted a more relaxed threshold of *P* < 5 × 10^−6^ for these factors. A total of 8 to 34 SNPs were identified as instrumental variables (Table S2, Supplemental Digital Content, http://links.lww.com/MD/N690). Utilizing the IVW method, the study highlighted suggestive associations between several cytokines and CHD risk. Notably, increased Eotaxin (CCL11) levels correlated with a heightened CHD risk, evidenced by an odds ratio (OR) of 1.10 and a 95% confidence interval (CI) of 1.03–1.18 (*P* = .003). This trend extended to other cytokines, including C–C motif chemokine ligand 20 (CCL20) (OR: 1.15, 95% CI: 1.05–1.25, *P* = .002), macrophage colony-stimulating factor 1 (CSF1) (OR: 1.09, 95% CI: 1.01–1.17, *P* = .020), Fibroblast growth factor 21 (FGF21) (OR: 1.14, 95% CI: 1.01–1.29, *P* = .038), Fms-related tyrosine kinase 3 ligand (FLT3LG) (OR: 1.26, 95% CI: 1.09–1.44, *P* = .001), and Neurotrophin-3 (NT-3) (OR: 1.12, 95% CI: 1.01–1.24, *P* = .026), all showing positive associations with CHD. Interestingly, an increase in leukemia inhibitory factor (LIF) levels was inversely associated with CHD risk (OR: 0.89, 95% CI: 0.80-0.99, *P* = .029). The primary findings of the MR analysis are displayed in Figure 2 and detailed in Table S3, Supplemental Digital Content, http://links.lww.com/MD/N690. In our MR study assessing CHD, the Cochran Q test detected no heterogeneity. The absence of a significant intercept suggests a lack of pleiotropy. Likewise, MR-PRESSO analysis indicated no horizontal pleiotropy (Table S4, Supplemental Digital Content, http://links.lww.com/MD/N690). Additionally, the robustness of our results is supported by a leave-one-out analysis, demonstrating no undue influence from any individual SNP, as elaborated in Figure 4.

### 3.2. Effects of CHD on 91 inflammatory cytokines

**Figure 3.**
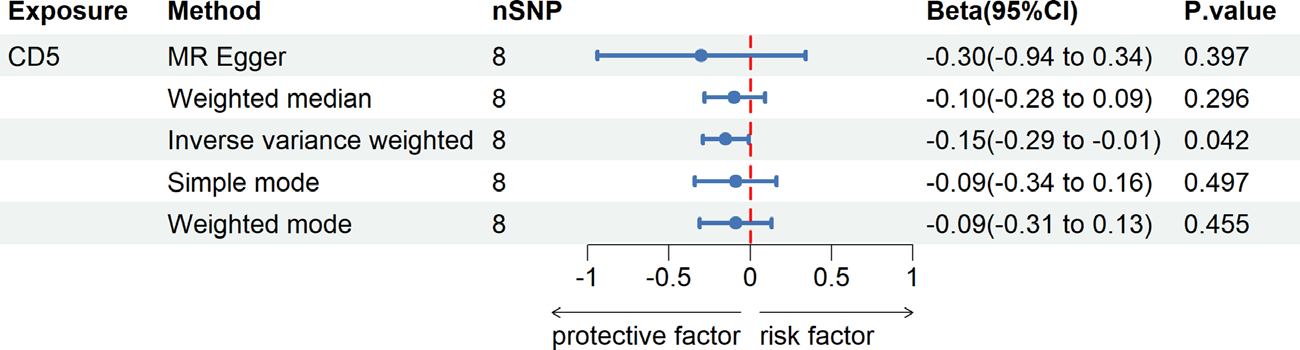
The causal effects of CHD on positive inflammatory cytokines using 5 MR methods. CD5 = T-cell surface glycoprotein CD5 levels.

**Figure 4.**
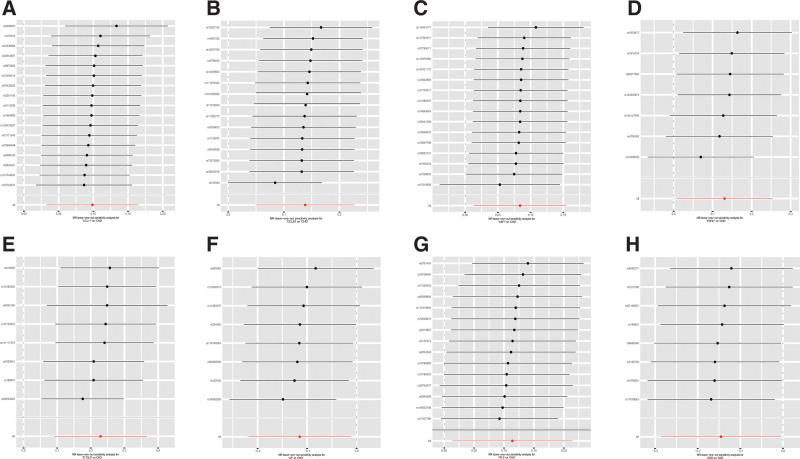
Forest plots depicting the causal relationship between CHD and 8 inflammatory cytokines – specifically CCL11 (A), CCL20 (B), CSF1 (C), FGF21 (D), FLT3LG (E), LIF (F), NT-3 (G), and CD5 (H) – are presented in the results of the “leave-one-out” analysis.

In this genetic study, we set a genome-wide significance threshold of 5 × 10^−8^. Following a comprehensive series of procedures, including linkage disequilibrium assessment, screening for SNPs with strong associations to eliminate confounders, and data harmonization, a total of 8 SNPs were ultimately identified as instrumental variables for our analysis (Table S5, Supplemental Digital Content, http://links.lww.com/MD/N690). Each of these SNPs exhibited an F-statistic value exceeding 10, suggesting a low probability of weak instrument bias, thereby enhancing the reliability of the results. The application of the IVW method yielded intriguing associations. Specifically, CHD showed a significant correlation with decreased levels of T-cell surface glycoprotein CD5 (CD5) (Beta: −0.15, 95% CI: −0.29 to −0.01, *P* = .042). These findings are comprehensively detailed in Figure 3 and Table S6, Supplemental Digital Content, http://links.lww.com/MD/N690, which also include results from the MR-Egger, weighted median, weighted mode analyses and simple mode analyses. Further bolstering the credibility of the study, the Cochran *Q* test revealed no significant heterogeneity, and the absence of a significant intercept in the MR-Egger analysis suggested a lack of pleiotropy. Complementing these findings, the MR-PRESSO analysis corroborated the absence of horizontal pleiotropy, thereby reinforcing the study’s methodological integrity. Table S7, Supplemental Digital Content (http://links.lww.com/MD/N690), succinctly summarizes the results of the pleiotropy and heterogeneity assessments. Additionally, the robustness of the MR analysis was affirmed through a “leave-one-out” analysis, as demonstrated in Figure 4. This analysis confirmed that no individual SNP unduly influenced the overall results, thereby attesting to the stability and reliability of the study’s conclusions.

## 4. Discussion

In our 2-sample MR analysis, we examined the causal links between 91 biomarkers, including chemokines, growth factors, interleukins, enzymes, interferons, tumor necrosis factors, receptors, and other cytokines as exposures, with CHD as the outcome. The study revealed that genetically predicted levels of CCL11, CCL20, CSF1, FGF21, FLT3LG, and NT-3 have a positive association with CHD risk, whereas LIF is negatively associated with it. When CHD was considered as the exposure in the MR, it suggestively may lead to decreased levels of CD5 through causal pathways. No reverse causalities were observed between any single biomarker and CHD. This implies that CCL11, CCL20, CSF1, FGF21, FLT3LG, and NT-3 may initiate CHD onset, while CD5 is likely downstream inflammatory regulators during disease progression.

Previous research on the relationship between CCL11 and CHD has yielded mixed outcomes. Certain studies have identified a correlation,^[28,29]^ whereas others have not observed any association.^[30,31]^ However, our study supports the connection between genetically predicted CCL11 levels and CHD, and its possible mechanism is that elevated CCL11 may exacerbate atherosclerotic plaque development by recruiting eosinophils and other immune cells, thus intensifying vascular inflammation and plaque instability. One study focused on the effects of CCL11 on human coronary artery endothelial cells. It found that CCL11 influences the expression of junctional molecules in these cells, impacting their monolayer permeability.^[32]^ The finding highlights the role of CCL11 in modulating endothelial cell function, which is critical in the development and progression of atherosclerosis. Additionally, the association of plasma CCL11 levels with the presence and extent of coronary atherosclerosis was examined in another study. This research adds to the growing body of evidence linking CCL11 to coronary artery disease, further elucidating its role in the pathophysiology of CHD.^[33]^ These studies collectively indicate that CCL11 plays a significant role in the mechanisms underlying CHD, particularly through its impact on endothelial cells and its association with myocardial infarction. Macrophage colony-stimulating factor (CSF1) is the main growth factor required to control the differentiation, survival, proliferation, and renewal of monocytes and macrophages.^[34]^ CSF1 plays a pivotal role in macrophage differentiation and survival.^[35]^ Studies have indicated that elevated circulating levels of CSF1 are linked to the progression of coronary atherosclerosis and could be a prognostic indicator.^[36,37]^ CSF1 contributes to atherogenesis and atherosclerosis by augmenting monocyte counts, enhancing monocyte migration to inflammatory sites, promoting macrophage differentiation, proliferation, and survival, and increasing lipid uptake by macrophages through the modulation of scavenger receptor expression levels.^[38]^ This evidence underscores the significant role of CSF1 in cardiovascular pathology. CCL20, a chemokine that attracts immature dendritic cells, effector/memory T cells, and naive B cells, has been observed at elevated levels in patients with atherosclerosis. Its presence in atherosclerotic lesions, identified as highly sensitive to inflammatory responses triggered by low-density lipoprotein, highlights its critical role in the inflammatory processes associated with coronary artery disease.^[39]^

Limited research on the relationship between FLT3LG and NT-3 and CHD. Previous research isn’t a direct study linking FLT3LG specifically to CHD mechanisms. However, FLT3LG is known to be involved in immune system regulation, particularly influencing T-cell activity. For instance, a study in the context of acute myelocytic leukemia showed that FLT3LG, along with IFITM3P6, correlates with T-cell activation in the bone marrow microenvironment and impacts patient survival. Although this study doesn’t directly relate to CHD, it highlights the importance of FLT3LG in immune cell regulation, which could be relevant in the broader context of inflammatory diseases, potentially including CHD.^[40]^ Further research is needed to establish a direct connection between FLT3LG and CHD. NT-3, secreted from human embryonic stem cell-derived cardiovascular progenitor cells (hCVPCs), contributes to cardiac repair by reducing cardiomyocyte death and enhancing angiogenesis. It acts by activating the extracellular signal-regulated kinase (ERK) and reducing the Bim level, leading to cardiac repair effects in infarcted hearts.^[41]^ There is limited research on the relationship between NT-3 and CHD, and our MR studies indicate that genetically predicted NT-3 is involved in the development of CHD.

LIF is an anti-inflammatory cytokine known for its affinity to the gp130/LIFR complex.^[42]^ It plays a critical role in moderating inflammation, primarily by fostering Treg differentiation while concurrently inhibiting Th17 cell differentiation.^[43]^ Studies have demonstrated LIF’s capacity to attenuate the progression of atherosclerosis, a finding that aligns with our research.^[44,45]^ This underscores the potential of LIF as a therapeutic target in the treatment and management of atherosclerosis-related diseases, including CHD. Fibroblast growth factor 21 (FGF21), a stress-responsive hormone, plays crucial roles in energy, glucose, and lipid regulation through the FGFR1 and β-klotho receptor complex.^[46]^ In our MR study, we have identified FGF21 as a potential risk factor for CHD. This finding stands in contrast to previous research that primarily highlighted the protective role of FGF21 in cardiovascular health, particularly through its regulation of lipid metabolism and inflammatory responses.^[47]^ Additionally, research has demonstrated the protective role of FGF21 in acute myocardial infarction, potentially through the activation of the AMPK-FGF21 loop, reducing ischemia/reperfusion injury in cardiac myocytes.^[48]^ However, our findings suggest that FGF21 may exhibit divergent roles, contributing to an increased risk of CHD under certain conditions. This discrepancy might be attributed to the complexity of FGF21’s mechanisms of action and its interactions with other metabolic and cardiovascular pathways. For instance, overexpression of FGF21 might lead to adverse cardiovascular outcomes in certain metabolic states, or FGF21’s role might vary across different types of CHD. Furthermore, genetic variability could influence individual responses to FGF21, leading to different disease risks across populations. In summary, our findings reveal the nuanced role of FGF21 in CHD, underscoring the need for further exploration of its mechanisms in future research. Moreover, these results suggest that therapeutic strategies targeting FGF21 should consider its potentially varying effects in different contexts.

In our MR study, CD5 was identified as a downstream factor associated with CHD. As a transmembrane protein pivotal in modulating T-cell-mediated immune responses, CD5’s direct connection with CHD presents a relatively unexplored facet in cardiovascular research.^[49]^ We hypothesize that CHD may influence T-cell functionality, thereby impacting the regulatory role of CD5 and potentially modifying T-cell subsets. This could result in altered CD5 expression. Moreover, CHD is characterized by systemic inflammation. This inflammatory state might cause alterations in lymphocyte populations, including those expressing CD5, thereby reducing its overall levels. CHD-induced physiological stress could suppress specific immune components like CD5-positive cells, potentially as a defensive response against inflammatory damage. Additionally, CHD-related endothelial dysfunction may affect immune cell behavior, including CD5 expression. Our study underscores a novel research path, emphasizing the need to discern whether the decrease of CD5 is a consequence or a contributory factor in CHD, and to elucidate the mechanisms underlying their interaction. Further exploration in this domain is vital for understanding the intricate inflammatory processes of CHD.

This study employs a bidirectional MR approach to examine the causal relationship between inflammatory factors and CHD, providing more robust evidence for the causal link between levels of inflammatory cytokines and the risk of CHD. Furthermore, we undertook an extensive review of pertinent literature to deepen our understanding of the connections among these factors. Unlike traditional observational studies, a significant advantage of this approach is its capacity to estimate causal relationships, effectively reducing concerns associated with reverse causation and confounding biases. Additionally, the use of genetic variants as instrumental variables strengthens the study’s independence, as these variants are generally unaffected by disease state or environmental factors. Advances in genomic technologies have made the genetic data we used more reliable and comprehensive, enhancing the study’s accuracy and scope. Moreover, MR study outcomes not only reveal potential biological mechanisms of disease but also help guide clinical prevention and treatment strategies, thus bolstering public health policy-making. However, it is crucial to acknowledge several inherent limitations in our MR study. Firstly, the inclusion of predominantly European-descended participants limits the broader applicability of our findings. Secondly, the link between inflammatory factors and CHD might be obscured by unidentified covariates. Lastly, unlike clinical trials,^[50,51]^ our study relied on aggregated data from large GWAS datasets, neglecting the analysis of stratified risk factors relevant to disease duration, severity, treatment approaches, and comorbidities. Therefore, future research should focus on validating our findings, which is essential for a deeper understanding of the complex relationship between inflammatory cytokines and CHD.

## 5. Conclusion

In this study, we employed MR analysis to provide additional insights into the relationship between inflammatory cytokines and CHD, suggesting that CCL11, CCL20, CSF1, FGF21, FLT3LG and NT-3 may be the upstream factors of CHD, while CD5 may be the downstream effect of CHD. Although the specific mechanisms of some inflammatory factors have not been fully elucidated, this study provides relevant clues, especially concerning FLT3LG, NT-3 and CD5 levels. This not only enhance our understanding of the complex molecular mechanisms underlying CHD but also open up new avenues for therapeutic intervention. Our study has established a foundational framework for future investigations, potentially paving the way towards reducing the incidence of CHD and enhancing patient prognoses.

## Acknowledgments

We extend our sincere thanks to all individuals and researchers who have contributed to this MR study.

## Author contributions

**Data curation:** Luo Lv, Yuli Guo.

**Methodology:** Zhongyi Zheng.

**Resources:** Luo Lv, Yuli Guo.

**Software:** Luo Lv, Yuli Guo.

**Visualization:** Luo Lv, Yuli Guo.

**Writing – original draft:** Luo Lv, Yuli Guo.

**Writing – review & editing:** Bao Li.

## Supplementary Material


